# Antimicrobial Activity Investigation on Wuyiencin Fractions of Different Polarity

**DOI:** 10.3390/molecules15053179

**Published:** 2010-04-29

**Authors:** Zengjie Cui, Kecheng Zhang, Gaimei She, Yinni Lin, Lei Sun, Yan Cheng, Beibei Tan

**Affiliations:** 1 State Key Laboratory for Biology of Plant Disease and Insect Pests, Institute of Plant Protection, Chinese Academy of Agricultural Sciences, Beijing 100193, China; 2 School of Chinese Pharmacy, Beijing University of Chinese Medicine, Beijing 100102, China; 3 Department of Biotechnology, Beijing City University, Beijing, 100094, China

**Keywords:** Wuyiencin, antimicrobial activity, *Rhodotorula rubra*, *Bacillus subtilis*, *Bacillus megaterium*, *Escherichia coli*, *Cladosporium fulvum*, *Staphylococcus aureus*

## Abstract

The aim of this study was to evaluate the antimicrobial activity of Wuyiencin fractions with different polarities against six indicator microorganisms: *Rhodotorula rubra*, *Bacillus subtilis*, *Bacillus megaterium*, *Escherichia coli*, *Cladosporium fulvum* and *Staphylococcus aureus*. The fermentation broth of Wuyiencin was submitted to AB-8 macroporous adsorptive resin and fractionated with solvents of different polarity. The fraction eluted with water had remarkably antimicrobial activity against all the microorganisms investigated except for *C. fulvum* and *S . aureus* (MIC ≤ 0.0625 mg/mL), probably due to the presence of active components. The fraction eluted with methanol showed potential antimicrobial activity against all the test microorganisms except for *R. rubra*, with MIC values of 0.5 and 2 mg/mL. In conclusion, fractions eluted with water and methanol, respectively, represent the main active-part of Wuyiencin, and could be emphasized for agricultural applications in the future.

## 1. Introduction

Biological pesticides are widely used to control plant diseases in the world today, and agricultural antibiotics played an important role in biological control. Wuyiencin is a secondary metabolite produced by *Streptomyces ahygroscopicus* var. wuyiensis which was isolated from Wuyi Mountain, Fujian Province of China in 1979, and identified in 1984 [1]. It is highly water soluble and as a pollution-free biological fungicide, it was characterized by high-efficiency, broad spectrum and low toxicity for the control crop fungi diseases, compared to other chemical pesticides [2]. Wuyiencin is effective in controlling crop diseases caused by fungi and bacteria, especially powdery mildew, gray mold, leaf mold and other fungal diseases. Furthermore, it also enhances crops’ resistance against diverse pathogens.

In view of these advantages, there has been a lot of research on Wuyiencin over the past 20 years. The titer of the wuyiencin producing strain was greatly improved by optimization of fermentation broth and protoplast fusion techniques [3]. The inhibition and the mode of action against *Botrytis cinerea* as well as other pathogens were characterized [4]. The absorbability of four carriers including attapulrite, kaolinite, diatomite and white carbon black has been tested in order to develop the best soluble powder formulation for Wuyiencin [5].

Wuyiencin has been produced at the factory scale and is commercialized in China to control fungal diseases of vegetable and field crops, such as tomato gray mold, powdery mildew in crops and so on. In order to extend the applications of Wuyiencin and improve the efficiency of utilization, our study was undertaken to evaluate the antimicrobial activity and search for the active constituents of wuyiencin by the agar diffusion assay against four gram-positive and gram-negative bacteria, one fungus and one yeast [6]. Preisolation of the fermentation broth was achieved through column chromatographic fractionation on AB-8 macroporous resin with water-methanol for gradient elution [7]. Fractions with different polarities showed inhibition against different indicator organisms. This study identified the fractions responsible for such antimicrobial activity and provided important evidence for the isolation and purification of active components in the future. 

## 2. Experimental

### 2.1. General

Wuyiencin fermentation broth was concentrated under vacuum at 40–45 ^o^C. AB-8 macroporous adsorptive resin (pore radius 130~140 A) was obtained from Tianjin Haiguang Chemical Factory, Tianjin, People’s Republic of China. All solvents were of analytical grade. 

### 2.2. Material

Wuyiencin fermentation broth was obtained from Weifang Wansheng Biopesticide Co., Ltd., Shandong Province in China, which is the only enterprise producing Wuyiencin. The test microorganisms used for antimicrobial testing included *Rhodotorula rubra*, *Bacillus subtilis*, *Bacillus megaterium*, *Escherichia coli*, *Cladosporium fulvum* and *Staphylococcus aureus*. The microorganisms were sourced from the State Key Laboratory for Biology of Plant Disease and Insect Pests, Institute of Plant Protection, Chinese Academy of Agricultural Sciences, P.R. China.

### 2.3. Fractionation

The concentrated fermentation broth was submitted to AB-8 macroporous adsorptive resin column chromatography, eluting with MeOH-H_2_O (0:1, 1:9, 3:7, 5:5, 7:3, 1:0), resulting in six fractions. One-column volume was used for each solution. Each fraction was collected, dried and assayed for antimicrobial activity.

### 2.4. Antimicrobial assays

Antimicrobial properties were estimated by the agar diffusion method [6]. The samples of the six fractions were freeze dried, and the samples were weighed and diluted in sterile MeOH:H_2_O (1:4) to obtain a concentration of 2 mg/mL. The stock solution was transferred into the next tube, and serial two-fold dilutions were prepared to give concentrations of 2, 1, 0.5, 0.25, 0.125, 0.0625 mg/mL [8,9]. The microorganism suspensions were finally diluted to 10^6^ CFU (colony forming units) mL^-1^ for use in the assays [8]. Plates containing MeOH:H_2_O (1:4) in medium served as solvent controls. The plates were incubated at 28 °C for 18h before the measurement of inhibition zones diameters. Minimal inhibitory concentration (MIC) was determined as the lowest concentration of the fractions able to completely inhibit visible growth of the microorganism, as detected by the naked eye [8,9]. Each test was performed in triplicate [10].

## 3. Results and Discussion

The fermentation broth of Wuyiencin was submitted to AB-8 macroporous adsorptive resin and fractionated with solvents of MeOH-H_2_O (0:1, 1:9, 3:7, 5:5, 7:3 and 1:0). Six fractions were collected, concentrated under vacuum, freeze dried and assayed for antimicrobial activity, respectively. 

In an antimicrobial test using the agar diffusion method [6], six Wuyiencin fractions were screened for their activity against six indicator microorganisms. Antimicrobial tests showed that fractions 1-6 exhibited antimicrobial effects against all the microorganisms tested. The MIC values of the test microorganisms to different fractions have been listed in Table 1.

**Table 1:** Antimicrobial activity of fractions 1-6 (MIC values, mg/mL).

	Microorganisms	fraction 1	fraction 2	fraction 3	fraction 4	fraction 5	fraction 6
*R. rubra*		0.0625	>2	>2	0.25	1	2
*B. subtilis*		<0.0625	>2	>2	>2	1	0.5
*B. megaterium*		<0.0625	0.5	0.5	>2	1	0.5
*E. coli*		<0.0625	>2	>2	>2	1	0.5
*C. fulvum*		0.5	-	-	-	-	0.5
*S. aureus*		-	-	-	-	-	0.5

**Figure 1:**
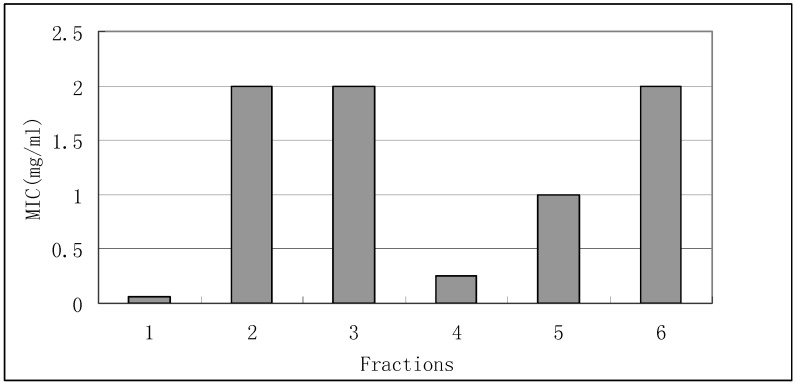
Antimicrobial activity of fractions 1-6 against *R. rubra*.

*R. rubra* is a yeast-like fungus of the *Cryptococ-caceae* family. *Rhodotorula* species are naturally spread in terrestrial and marine materials, such as soil, water, plants, and animal residues [11]. They may colonize plants, humans, and other mammals. The antimicrobial activity assays in our lab have previously indicated that wuyiencin showed potential inhibitory effects against *R. rubra* in comparison with most fungal pathogens found in agriculture under the same culture condition, so *R*. *rubra* was chosen as the representative of agricultural fungal pathogens. The antimicrobial results showed that fractions 1 and 4 possessed potential antimicrobial inhibition, with MIC values of 0.0625 and 0.25 mg/mL, respectively. Fractions 2, 3, 5 and 6 showed higher MIC values and lower antimicrobial activity (Figure 1).

**Figure 2:**
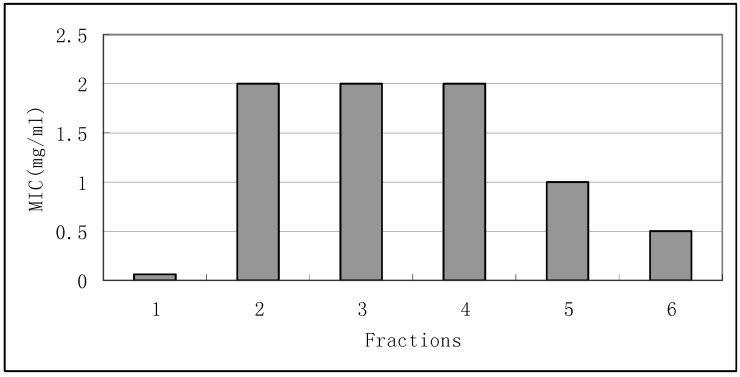
Antimicrobial activity of fractions 1-6 against *B. subtilis*.

*B. subtilis*, known as the hay bacillus or grass bacillus, is a gram-positive, catalase-positive bacterium commonly found in soil [12]. *B. subtilis* is not considered a human pathogen; it may contaminate food but rarely causes food poisoning [13]. It was usually used as bacterial indicator organism. Results showed potential inhibition for fractions 1 and 6 against *B. subtilis* with MIC values of 0.0625 and 0.5 mg/mL. Fractions 2, 3, 4, and 5 were found to have lower antimicrobial activity (Figure 2).

**Figure 3:**
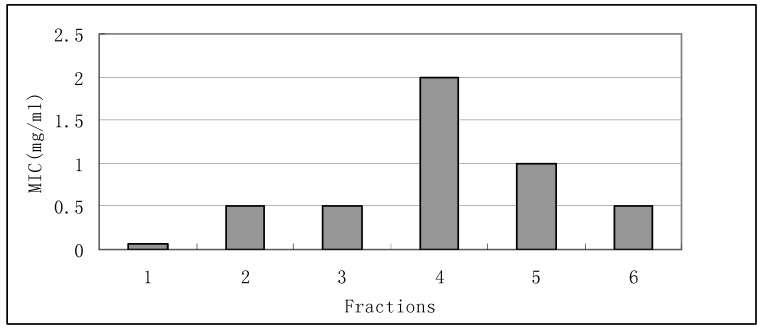
Antimicrobial activity of fractions 1-6 against *B. megaterium*.

*B. megaterium* is a rod-shaped, gram-positive, endospore forming, species of bacteria used as a soil inoculant in agriculture and horticulture [14]. The results showed that fraction 1 also possessed great antimicrobial activity against *B. megaterium*. Fractions 2, 3 and 6 showed similar antimicrobial activity against *B. megaterium* with MIC values of 0.5 mg/mL (Figure 3).

**Figure 4:**
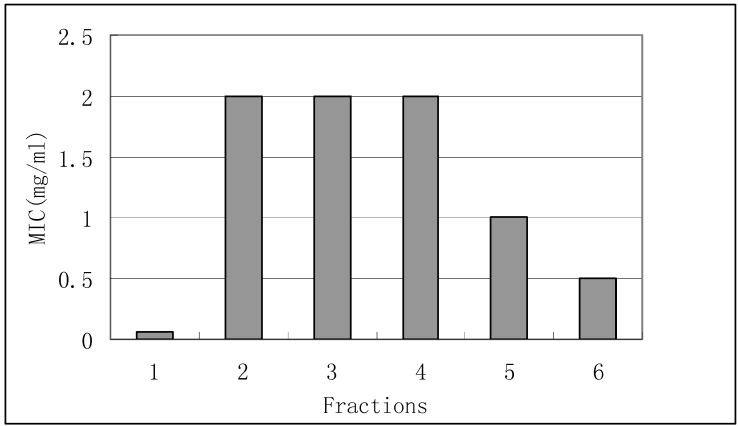
Antimicrobial activity of fractions 1-6 against *E. coli*.

Most *E. coli* strains are harmless, but some can cause serious food poisoning in humans, and are occasionally responsible for product recalls [15]. *E. coli* are not always confined to the intestine, and their ability to survive for brief periods outside the body makes them an ideal indicator organism to test environmental samples for fecal contamination [16]. Results showed potential inhibition for fractions 1 and 6 against *E. coli*, with MIC values of 0.0625 and 0.5 mg/mL. Fractions 2, 3, 4 and 5 showed little inhibition effect with MIC values of 2 and 1 mg/mL (Figure 4).

Because of their short growing phase, *E. coli*, *B. subtilis*, *B*. *megaterium* can divide and form a colony of hundreds of bacteria in just a few hours under a suitable environment. Their ability to survive for brief periods makes them ideal indicator organisms.

*C*. *fulvum* is a specialized, biotrophic pathogen, which causes leaf mold on tomato [17]. *C. fulvum* has been a serious disease of glasshouse tomatoes all over the world. It is also important on the field crop where conditions of high humidity prevail. The disease can cause serious losses in the field or in glasshouses [18]. Results showed potential inhibition for fractions 1 and 6 against *C*. *fulvum*, with MIC values of 0.5 mg/mL.

*S. aureus* is one of the commonest and most important gram-positive hospital-acquired organisms. It has a high propensity to colonize abnormal skin surfaces and open wounds. *S. aureus* can cause a range of illnesses from minor skin infections, such as pimples, impetigo, to life-threatening diseases such as pneumonia, meningitis. *S. aureus* remains one of the five most common causes of nosocomial infections, often causing postsurgical wound infections [19]. The results mentioned above results showed potential inhibition for fraction 6 against *S*. *aureus*, with MIC value of 0.5 mg/mL.

In conclusion, antimicrobial tests revealed that fraction 1 of Wuyiencin possessed greater antimicrobial activity (MIC ≤ 0.0625 mg/mL) against all the microorganisms investigated. Fraction 6 showed potential antimicrobial activity against all the microorganisms except for *R. rubra*, with MIC value of 0.5 mg/mL. Fractions1 and 4 were found to be more effective against *R. rubra*, which indicated that they might contain compounds active against agricultural pathogens. All the tested microorganisms except for *C. fulvum* and *S. aureus* were less sensitive to fraction 5 with the MIC of 1 mg/mL. The results implied that fraction 1 could contain highly active components against pathogens, and fraction 6 against some bacteria.

## Conclusions

This study indicated macroporous adsorptive column chromatography is an effective method to preisolate the active components of Wuyiencin. Antimicrobial activity results showed that these fractions have different antimicrobial activity. Especially, fraction 1, eluted with water, was found to be the most effective against all the indicator microorganisms except for *S. aureus*, which provided useful information for the next work to isolation and purification of active components. Fraction 6 showed potential activity against all the microorganisms with MIC values of 0.5 and 2 mg/mL. The study indicated the possibility for different fractions of Wuyiencin to be exploited as antimicrobial agents to control different crop diseases, thus the efficiency of utilization for Wuyiencin will be improved and the applications in agriculture extended.

## References

[1] Wei R.Q., Lin D.C., Chen Z.H. (1984). Identification of the producing strain of agricultural antibiotic Bo-10. Acta Microbiol. Sin..

[2] Zeng H.M., Shi Y.P. (2003). Wuyiencin, a new antibiotic for controlling crop Fungi diseases. Fine and Specialty Chemicals.

[3] Zeng H.M., Zhang Z.L., Shi Y.P., Lin D.X. (1995). Enhancing the production of Wuyiencin through protoplast fusion. Acta Microbiol. Sin..

[4] Sun Y.Z., Zeng H.M., Shi Y.P., Li G.Q. (2003). Mode of action of Wuyiencin on *Botrytis cinerea*. Acta Phytipathol. Sinica.

[5] Feng C., Zhang K.C., Yuan H.Z., Yang D.B., Wang P., Sun L.P. (2008). The formulation of Wuyiencin soluble powder. Chin. J. Biol. Control.

[6] Daljit S.A., Jasleen K. (1999). Antimicrobial activity of spices. Int. J. Antimicrobial Agents.

[7] Mohamed S.A., Marzouk. (2008). An acylated flavonol glycoside and hydrolysable tannins from *Callistemon lanceolatus* flowers and leaves. Phytochem. Analysis.

[8] Begnami A.F., Duarte M.C.T., Furletti V., Rehder V.L.G. (2010). Antimicrobial potential of *Coriandrum sativum* L. against different *Candida* species *in vitro*. Food Chem.

[9] Mohamed A.A.O. (2010). Synthesis and antimicrobial activity of new 5-(2-Thienyl)-1,2,4-triazoles and 5-(2-Thienyl)-1,3,4-oxadiazoles and related derivatives. Molecules.

[10] Zhang Y., Mu J., Gu X.J., Zhao C.Y., Wang X.L., Xie Z.P. (2009). A marine sulfate-reducing bacterium producing multiple antibiotics: Biological and chemical investigation. Marine Drugs.

[11] Samonis G., Anatoliotaki M., Apostolakou H., Maraki S., Mavroudis D., Georgoulias V. (2001). Transient fungemia due to *Rhodotorula Rubra* in a Cancer Patient: Case Report and Review of the Literature. Infection.

[12] Madigan M., Martinko J. (2005). Brock biology of microorganisms.

[13] Ryan K.J., Ray C.G. (2004). Sherris Medical Microbiology.

[14] Rajendran G., Sing F., Desai A.J., Archana G. (2008). Enhanced growth of pigeon pea by co-inoculation of *Bacillus* strains with *Rhizobium* spp. Bioresource Technol..

[15] Vogt R.L., Dippold L. (2005). Escherichia coli O157:H7 outbreak associated with consumption of ground beef, June-July 2002. Public Health Rep..

[16] Winfield M.D., Groisman E.A. (2003). Role of nonhost environments in the lifestyles of *Salmonella* and *Escherichia coli*. Appl. Environ. Microbiol..

[17] Peter E.H., John W.K., Bolton M.D. (2008). The *Cladosporium fulvum* Virulence Protein Avr2 Inhibits Host Proteases Required for Basal Defense. Plant Cell.

[18] Curtis M.D., Gore J., Oliver R.P. (1994). The phylogeny of the tomato leaf mould fungus *Cladosporium fulvum* syn. *Fulvia fulva* by analysis of rDNA sequences. Curr. Genetics.

[19] Kluytmans J., Belkum A., Verbrugh H. (1997). Nasal carriage of *Staphylococcus aureus*: epidemiology, underlying mechanisms, and associated risks. Clin. Microbiol. Rev..

